# Patientenspezifische 3D-Druck-Implantate und -Schablonen für Ellenbogen und Unterarm

**DOI:** 10.1007/s00113-025-01548-z

**Published:** 2025-03-03

**Authors:** Christopher Cramer, Samuel Sperlich, Annika Hättich, Tobias Dust, Karl-Heinz Frosch, Konrad Mader

**Affiliations:** Klinik und Poliklinik für Unfallchirurgie und Orthopädie, Sektion Hand‑, Unterarm- und Ellenbogentraumatologie, Universitätsklinikum Hamburg-Eppendorf (UKE), Martinistraße 52, 20246 Hamburg, Deutschland

**Keywords:** Malunionen, Kongenitale Deformitäten, Tumorbedingte Deformitäten, Korrekturosteotomie, Computer-aided design, Malunited fracture, Congenital deformities, Tumor-related deformities, Corrective osteotomy, Computer-aided design

## Abstract

Der 3D-Druck hat Einzug in die Orthopädie und Unfallchirurgie gehalten, insbesondere bei komplexen Eingriffen an Ellenbogen und Unterarm. Durch die Herstellung patientenspezifischer Implantate und chirurgischer Schablonen können Fehlstellungen, Frakturen und Deformitäten präzise korrigiert werden. Es konnte gezeigt werden, dass diese Technologie die Operationsgenauigkeit erhöht, die Genesungszeit verkürzt und postoperative Komplikationen reduziert. Die 3D-gedruckten Implantate bieten gegenüber herkömmlichen Implantaten den Vorteil der individuellen Anpassung an die anatomische Situation des Patienten. Dies ist besonders relevant in komplexen Fällen wie Malunionen, angeborenen Fehlbildungen (z. B. Madelung-Deformität) und tumorbedingten Deformitäten. Die präoperative Planung mit 3D-Modellen ermöglicht eine detaillierte Simulation des Eingriffs und optimiert die Platzierung der Implantate. Trotz der Vorteile bleiben Herausforderungen bestehen: Die Herstellung patientenspezifischer Implantate ist zeit- und kostenintensiv. Zudem erfordert die Technologie Expertise und spezielle Ressourcen, was ihre Anwendung in weniger spezialisierten Zentren limitiert. Zukünftige Entwicklungen, wie der 4D-Druck mit formverändernden Implantaten, versprechen weitere Fortschritte. Durch die Kombination von Präzision, Individualisierung und dynamischer Anpassungsfähigkeit könnten 3D-gedruckte Implantate die Patientenversorgung in der Orthopädie und Unfallchirurgie nachhaltig verbessern.

**Video online:**

Die Online-Version dieses Beitrags (10.1007/s00113-025-01548-z) enthält ein zusätzliches Video, das eine Korrekturosteotomie am distalen Unterarm zeigt.

## Abstract (en)

The process of 3D printing has found its way into orthopedics and trauma surgery, particularly for complex interventions on the elbow and forearm. By producing patient-specific implants and surgical templates misalignments, fractures and deformities can be precisely corrected. It could be shown that this technology increases the surgical accuracy, shortens recovery times and reduces postoperative complications. Compared to conventional implants 3D-printed implants provide the advantage of individual adaptation to the anatomical situation of the patient. This is particularly relevant in complex cases, such as malunions, congenital malformations (e.g., Madelungʼs deformity) and tumor-related deformities. The preoperative planning with 3D models enables a detailed simulation of the procedure and optimizes the placement of the implants. Despite these advantages challenges still remain: the production of patient-specific implants is time-consuming and cost-intensive. In addition, the technology requires expertise and special resources, which limit its application in less specialized centers. Future developments, such as 4D printing with shape-changing implants, promise further progress. By combining precision, individualization and dynamic adaptability, 3D-printed implants could sustainably improve patient care in orthopedics and trauma surgery.

Die Entwicklung medizinischer Technologien hat in den letzten Jahrzehnten zu signifikanten Verbesserungen in der Präzision, Sicherheit und Effizienz komplexer chirurgischer Eingriffe geführt. Eine der wegweisendsten Innovationen in der Orthopädie und Unfallchirurgie ist die Einführung der 3D-Planung und des 3D-Drucks, insbesondere für die Herstellung von patientenspezifischen Instrumenten und Implantaten. Der Ellenbogen und der Unterarm sind aufgrund ihrer komplizierten anatomischen Struktur sowie der Vielzahl an Bewegungsachsen besonders anfällig für Frakturen, Fehlstellungen und Deformitäten. Die Vielzahl der Bewegungsachsen sowie die enge Verzahnung von Gelenkflächen, Bändern und Muskeln stellen eine erhebliche Herausforderung für herkömmliche chirurgische Ansätze dar. Solche Eingriffe erfordern ein hohes Maß an Expertise und Planung. Patientenspezifische 3D-gedruckte Implantate bieten die Möglichkeit, maßgeschneiderte Lösungen, die präzise an die individuellen anatomischen Gegebenheiten eines Patienten angepasst sind und die chirurgischen Ergebnisse verbessern können, zu entwickeln [1, 2].

## Hintergrund und Relevanz des Themas

Die Relevanz des Themas ist in den letzten Jahren deutlich gestiegen, da gezeigt werden konnte, dass 3D-gedruckte Implantate postoperative Komplikationen verringern und die Genesungszeit verkürzen können [3]. Dies ist bedingt durch die akkurate Platzierung auf deformierten Knochen sowie das bessere visuelle und haptische Feedback in der Operation [4–9].

Zu diesen Vorteilen kommt die Möglichkeit, die Operation präzise, 3D-gestützt zu planen und Instrumente anzupassen, was mit einer besseren Passform der Implantate sowie einer Verkürzung der Operationszeit, kürzerer intraoperativer Durchleuchtungszeit, weniger Blutverlust und einer verbesserten postoperativen Beweglichkeit einhergeht [10].

Mithilfe des 3D-Drucks können hochkomplexe, patientenspezifische Implantate hergestellt werden

Der 3D-Druck hat sich von einer Prototyping-Technologie zu einer etablierten Methode entwickelt, die in der Lage ist, hochkomplexe, patientenspezifische Implantate herzustellen. Fortschritte in der Materialwissenschaft und der Fertigungstechnologie haben es ermöglicht, Implantate aus biokompatiblen Materialien wie Grade-5-Titan, Polyethylenen und speziell beschichteten Kunststoffen zu drucken; diese weisen eine hohe Festigkeit und gute biologische Verträglichkeit auf [11, 12]. Die präoperative Planung profitiert ebenfalls stark vom Einsatz der 3D-Visualisierungstechniken. Diese Technologien ermöglichen es, ein exaktes digitales Modell des betroffenen Knochens zu erstellen sowie die Planung und Anpassung des Implantats zu optimieren. Diese präoperative Vorbereitung erhöht die intraoperative Sicherheit, indem sie den Einsatz patientenspezifischer Instrumente, die eine präzise Führung bieten, ermöglicht [11]. Die Anwendung von 3D-gedruckten Modellen erlaubt auch eine präzisere Darstellung der pathologischen Veränderung. Dies führt zu einem besseren Verständnis, von dem insbesondere weniger erfahrene Chirurgen/-innen profitieren [2].

## Herstellung und Implantation

Die Genauigkeit des Druckverfahrens spielt bei der Herstellung eine entscheidende Rolle. Wong et al. betonten, dass poröse Strukturen die Integration in das umgebende Knochengewebe verbessern und die Langzeitstabilität erhöhen [13]. Diese Strukturen fördern die Einheilung und reduzieren das Risiko einer Lockerung. Dies ist insbesondere für die Langzeitstabilität von Vorteil. Eine sorgfältige Nachbearbeitung, wie die Sterilisation und die Anwendung antibakterieller Beschichtungen, ist entscheidend, um Infektionsrisiken zu reduzieren und die Biokompatibilität zu verbessern [12].

## Spezielle Anwendungsfälle und Korrekturosteotomie

Die 3D-gedruckten Implantate im Bereich des Ellenbogens und Unterarms sind besonders bei komplexen Frakturen, posttraumatischen Deformitäten, angeborenen Fehlbildungen, Rekonstruktionen nach Tumorresektionen sowie bei Rotations- und Achsfehlstellungen indiziert. Sie ermöglichen die präzise Anpassung an die individuelle anatomische Situation und eine maßgeschneiderte Gestaltung, die mit herkömmlichen Methoden nur schwer oder gar nicht zu erreichen ist.

### Korrekturosteotomie

Korrekturosteotomien sind chirurgische Eingriffe, die u. a. zur Behandlung von Malunionen eingesetzt werden. Malunionen können funktionelle und ästhetische Beeinträchtigungen sowie Schmerzen verursachen. Gemäß Saravi et al. wird dieser Eingriff insbesondere bei Fehlstellungen der oberen Extremitäten angewendet [14]. Solche Fehlstellungen können nach konservativer oder operativer Frakturbehandlung auftreten und das Arthroserisiko erhöhen. Eine Korrekturosteotomie ist indiziert bei signifikanter Bewegungseinschränkung, Schmerzen oder sichtbaren Deformitäten. Die Autoren betonten, dass die präoperative 3D-Planung und patientenspezifische chirurgische Schablonen eine präzise Rekonstruktion der anatomischen Struktur ermöglichen sowie zu einer erheblichen Verbesserung der Beweglichkeit und Schmerzreduktion führen.

### Angeborene Fehlstellungen und spezifische Deformitäten

Angeborene Fehlstellungen der oberen Extremitäten, wie kongenitale radioulnare Synostosen und die Madelung-Deformität, stellen oft eine chirurgische Herausforderung dar, insbesondere wenn Standardimplantate den individuellen Bedürfnissen des Patienten nicht gerecht werden. In solchen Fällen bieten 3D-gedruckte Implantate eine vielversprechende Lösung. Choo konnte zeigten, dass diese maßgeschneiderten Implantate eine präzise Anpassung an die anatomische Einzigartigkeit des Patienten erlauben sowie mit einer verbesserten Heilung und Funktion assoziiert sind [15]. Durch die 3D-Planung und die Herstellung patientenspezifischer Implantate können komplexe Mehrebenendeformitäten effizient mit einer einzigen Osteotomie korrigiert werden. Diese Technologie trägt dazu bei, den Bedarf an herkömmlichen Knochentransplantaten zu reduzieren sowie gleichzeitig die Präzision und das Ergebnis der Korrektur zu optimieren.

### Madelung-Deformität

Die Madelung-Deformität ist eine komplexe angeborene Fehlstellung des Unterarms, die durch ein gestörtes Wachstum des volarulnaren Teils der distalen Radiusepiphyse verursacht wird. Dies resultiert in einer charakteristischen Krümmung des Knochens. Bachy et al. beschrieben die Verwendung von 3D-gedruckten, patientenspezifischen Schnittführungen zur Korrektur dieser Fehlstellung [16]. Präoperative CT-Scans wurden zur Planung der Osteotomien genutzt. Diese maßgeschneiderten Schnittführungen ermöglichten präzise Korrekturen des deformierten Radius in mehreren Ebenen, was die klinischen und radiologischen Ergebnisse verbesserte. Die Autoren betonen, dass diese innovative Methode die Präzision der Operation steigert und intraoperative Risiken minimiert.

Die Studie umfasste 8 Patienten mit schwerer Madelung-Deformität, die mithilfe von 3D-geplanten, maßgeschneiderten Schnittführungen operiert wurden. Die präoperativen Durchschnittswerte des radialen Bogens betrugen 32° (a.-p.) und 36° (seitlich). Nach der Korrektur in Form von Doppelosteotomien verbesserten sich diese Werte auf durchschnittlich 10° (a.-p.) und 7° (seitlich). Diese Ergebnisse verdeutlichen die Effektivität von 3D-geplanten Verfahren bei komplexen Deformitäten.

### Posttraumatische Deformitäten

Posttraumatische Deformitäten entstehen, wenn Frakturen des Unterarms oder der distalen Radiusregion fehlerhaft verheilen. Dies löst funktionelle Einschränkungen und Schmerzen aus, da die Biomechanik der distalen Radioulnar- und Radiokarpalgelenke beeinträchtigt wird. Schindele et al. untersuchten die Anwendung von 3D-geplanten, patientenspezifischen Implantaten und Instrumenten für die chirurgische Korrektur solcher Deformitäten [5]. Die Autoren beschreiben, dass die präzise 3D-Planung und der Einsatz von „patient-specific implants“ und „patient-specific instruments“ eine individuelle Anpassung an die spezifische Anatomie des Patienten ermöglichten. Dies führte zu einer exakten Platzierung der Platten, insbesondere in schwer zugänglichen Bereichen wie der Diaphyse, in denen herkömmliche Platten schwierig zu positionieren sind. Durch diese Methodik konnten postoperative funktionelle und radiologische Verbesserungen erzielt werden, indem die Ausrichtung der Knochen an die unverletzte Seite angeglichen wurde.

#### Fallbeispiel 1

**Abb. 1:**
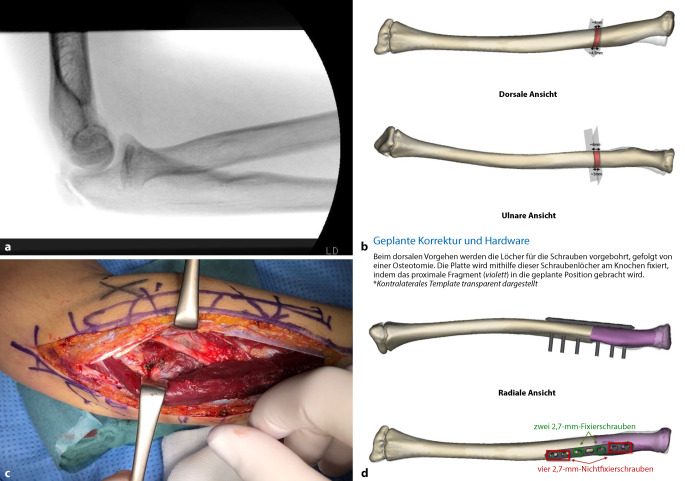
**a** Intraoperative Darstellung des Ellenbogengelenks im seitlichen Strahlengang. *Beachte* die falsche Projektion des Radiusschafts in Relation zum Epicondylus radialis, **b** präoperative Planung der Osteotomie und knöchernen Resektion. (Planung durch Fa. Materialise, Leuven, Belgien), **c** intraoperative Darstellung des proximalen Radius mit freigelegtem N. radialis, **d** präoperative Planung der geschlossenen Osteotomie und Plattenosteosynthese

Ein 14-jähriger Patient stellte sich in unserer Spezialsprechstunde für Ellenbogenverletzungen mit einer bisher nichtdiagnostizierten, veralteten Radiushalsfraktur vor. Diese hatte eine chronische Subluxation des Radiuskopfes, die mit einer Einschränkung der Supination einherging, zur Folge. Radiologisch zeigte sich ein dezentrierter Radiuskopf. Klinisch imponierte die eingeschränkte Supination bei einer Pronation von 80°. Ein Flexionsdefizit lag nicht vor. Mit der Familie wurde die Möglichkeit einer Korrekturosteotomie nach vorheriger 3D-Planung der Deformität besprochen. Die computerunterstützte 3D-Planung ergab, dass eine Umstellungsosteotomie sowohl des proximalen Radius als auch der Ulna mit additiver Plattenosteosynthese erforderlich war. Der Patient stellte sich nach 6 und 12 Wochen erneut in unserer Sprechstunde vor. Radiologisch zeigte sich eine zunehmende knöcherne Konsolidierung bei deutlicher Verbesserung der Supination. Anhand dieses Fallbeispiels wird der positive Effekt millimetergenauer computergestützter Planung auf die intraoperativen Abläufe besonders deutlich (Abb. 1 und 2).

**Abb. 2:**
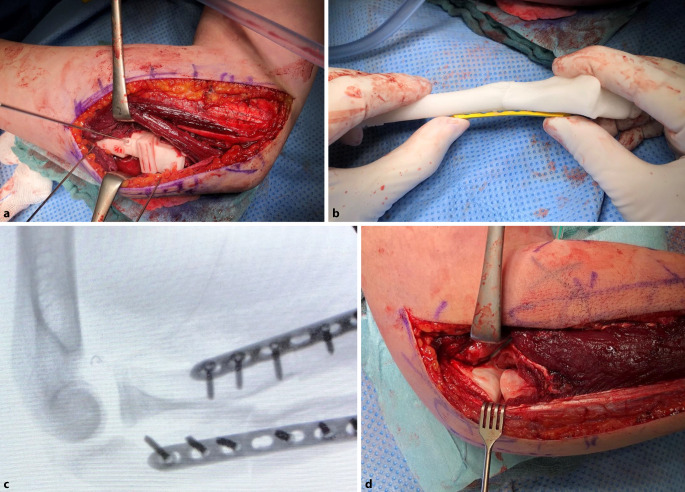
**a** Intraoperative Präparation der proximalen Ulna, bereits aufgesetzte 3D-gedruckte Sägelehre. **b** Die definitive Platte kann ex situ modelliert werden. **c** Intraoperative seitliche Darstellung des Ellenbogengelenks mit nun korrekt zentriertem Radiusschaft, **d** anatomische Darstellung des rezentrierten Radiuskopfes

#### Fallbeispiel 2

**Abb. 3:**
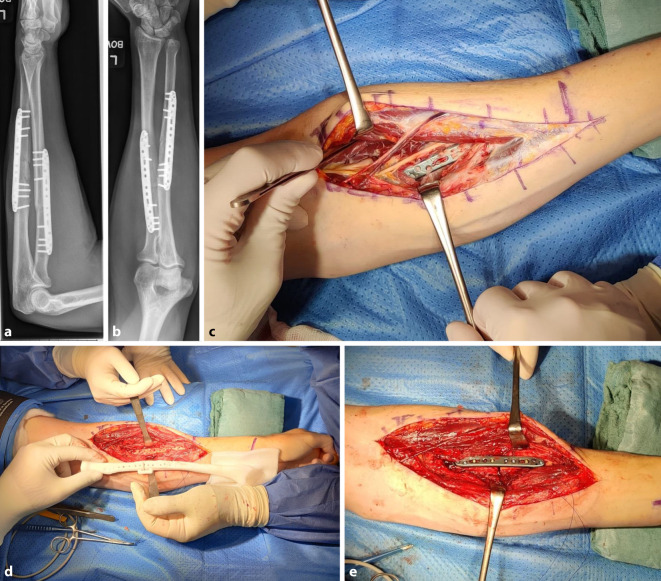
**a, b** Osteosynthetisch versorgte Unterarmschaftfraktur. Die Fraktur ist noch nicht vollständig konsolidiert. **c** Plattenlage in Relation zum Verlauf des N. radialis. **d** Positionierung der Bohrlöcher zur Ermöglichung der korrekten Torsion des Radius. **e** Einliegende Platte nach komplettierter Osteosynthese und Drehung des Radiusschaftes

Ein 43-jähriger Mann stellte sich in unserer Sprechstunde knapp 6 Monate nach operativ versorgter Unterarmfraktur mit vollständigem Verlust der Pronationsfähigkeit des Unterarms vor. Die Supinationsbewegungen waren frei, die Extension und Flexion ebenfalls. Die klinische Untersuchung ergab einen intakten N. radialis. In der durchgeführten bildgebenden Röntgen- und CT-Untersuchung konnten eine Torsionsfehlstellung sowie eine fehlverheilte Fraktur des Radius diagnostiziert werden. Anhand einer präoperativen 3D-Bildgebung wurde die Korrektur der Fehlstellung geplant. Diese umfasste eine Osteotomie des Radius sowie eine intraoperative Derotation zur Wiederherstellung der Pronationsfähigkeit. Intraoperativ wurde ein freier Bewegungsumfang erreicht. Dieser blieb auch bei den Nachuntersuchungen nach 3 und 6 Monaten erhalten (Abb. 3).

### Distale Radiusfraktur

Distale Radiusfrakturen sind häufige Verletzungen, die präzise chirurgische Eingriffe erfordern. Kong et al. nutzten die 3D-Drucktechnik, um vor der Operation ein physisches Modell des Frakturbereichs zu erstellen. Dies ermöglichte eine detaillierte Operationssimulation, die mit reduzierter Operationszeit, weniger Blutverlust und weniger intraoperativen Durchleuchtungen einherging [17]. Allerdings gab es keine signifikanten Unterschiede in den postoperativen Ergebnissen (Beweglichkeit und Schmerzbewertung) zwischen der 3D- und der Routine-Gruppe.

Schindele et al. untersuchten den Einsatz von 3D-gedruckten, patientenspezifischen Platten zur Behandlung von posttraumatischen Frakturen des distalen Radius. Die Ergebnisse zeigten, dass die Verwendung solcher individuell angepassten Implantate die Knochenheilung verbesserte und die Patientenzufriedenheit steigerte [5]. Ähnlich wie bei Kong et al. ermöglichte die präoperative Planung mittels 3D-Visualisierung eine genaue Bestimmung der Frakturstellen und eine präzise Anpassung der Platten. Dies spiegelte sich in einer reduzierten Operationsdauer und einer schnelleren postoperativen Rehabilitation wider.

### Tumorbedingte Deformitäten

Tumorbedingte Deformitäten, insbesondere im Bereich des proximalen Radius, stellen eine besondere Herausforderung für die rekonstruktive Chirurgie dar. Der Verlust dieser anatomisch wichtigen Struktur kann erhebliche funktionelle Einschränkungen, wie verminderte Stabilität und Beweglichkeit in Ellenbogen und Unterarm, auslösen, insbesondere wenn die Tumoren die epiphysäre Region betreffen. Der Einsatz von 3D-gedruckten, personalisierten Prothesen erlaubt eine präzise Anpassung an die individuelle anatomische Situation des Patienten und eine anatomiegerechte Wiederherstellung der Gelenkstruktur. Li et al. berichten, dass die Patienten eine signifikante Verbesserung der Bewegungsreichweite und Kraft nach der Operation erreichten, was eine zufriedenstellende Funktionswiederherstellung unterstützte [18].

Der Vorteil dieser personalisierten Prothesen liegt in ihrer Fähigkeit, eine verbesserte Osseointegration zu fördern, was zu Stabilität und Langlebigkeit der Implantate beiträgt. Die Autoren betonten, dass die Verwendung poröser Oberflächen, die das Knochenwachstum begünstigen, keine Komplikationen wie aseptische Lockerung oder Infektionen bedingen. Dies ist ein entscheidender Faktor, um die langfristige Funktion und Stabilität der rekonstruierten Struktur sicherzustellen. Die Studie zeigt, dass 3D-gedruckte, personalisierte Prothesen eine vielversprechende Lösung zur Behandlung von tumorbedingten Deformitäten des proximalen Radius bieten. Die präzise Anpassung an die individuelle anatomische Situation und die Unterstützung der Osseointegration machen diese Technologie zu einer wertvollen Alternative in der rekonstruktiven Chirurgie, die postoperative funktionelle Ergebnisse erheblich verbessern kann.

## Diskussion und Ausblick

Jüngere Studien zeigen deutlich, dass der Einsatz von 3D-gedruckten, patientenspezifischen chirurgischen Modellen und Implantaten bei der Korrektur von fehlverheilten diaphysären Unterarmfrakturen deutliche Vorteile bietet.

Wahl des Schablonentyps und Passgenauigkeit je nach anatomischer Region sind entscheidend

Es konnte festgehalten werden, dass durch präoperative 3D-Planung und die Anwendung patientenspezifischer chirurgischer Schablonen eine präzise Durchführung komplexer, multiplanarer Korrekturosteotomien möglich ist [1]. Diese Methode führte zu signifikanten Verbesserungen der Bewegungsreichweite, Griffkraft und Schmerzlinderung. Die Korrekturen entsprachen nahezu exakt den geplanten präoperativen Simulationen.

Caiti et al. untersuchten die Positionierungsgenauigkeit von 3D-gedruckten, patientenspezifischen chirurgischen Schablonen für den Radius. Sie zeigten, dass sowohl der anatomische Anbringungsort als auch das Design der Schablone die Präzision der Platzierung erheblich beeinflussen [19]. Die Studie ergab, dass Schablonen im distalen und im proximalen Bereich des Radius eine signifikant höhere Positionierungsgenauigkeit aufweisen. Im mittleren Diaphysenbereich kam es jedoch zu einer erhöhten Rotationsfehlerrate, was auf die eingeschränkte Verankerung der Führungen in dieser Zone zurückzuführen ist. Diese Ergebnisse verdeutlichen, dass die Wahl des Schablonentyps und die Passgenauigkeit in Abhängigkeit von der anatomischen Region entscheidend für den Erfolg der Operation sind. Die Komplexität und der erhebliche Ressourcenaufwand bei der Planung und Herstellung dieser patientenspezifischen Instrumente können jedoch ihre Anwendung in weniger spezialisierten chirurgischen Einrichtungen limitieren.

Luenam et al. beschrieben, dass der Einsatz von 3D-gedruckten, patientenspezifischen Titanimplantaten in der Behandlung distaler Humerusfrakturen eine therapeutische Alternative darstellen kann [20]. Solche Implantate bieten durch ihre präzise anatomische Passform und optimierte Biokompatibilität eine höhere Stabilität und Funktionalität, was mit einer verbesserten Langzeitprognose einhergeht. Im vorgestellten Fall konnte durch die Verwendung eines individualisierten, mithilfe des 3D-Drucks hergestellten Implantats nicht nur die Knochenrekonstruktion, sondern auch die Refixation des lateralen Kollateralbandkomplexes gewährleistet werden. Ein limitierender Faktor bleibt jedoch die zeitintensive Herstellung und Planung dieser patientenspezifischen Prothesen, die besonders in akuten und dringenden chirurgischen Szenarien eine Herausforderung darstellt.

Patientenspezifische 3D-Druck-Implantate schaffen einen signifikanten Mehrwert

Labèr et al. untersuchten die Anwendung von 3D-Planungen zur Korrektur distaler Radiusfehlstellungen [10]. Sie zeigten, dass patientenspezifische Instrumente (PSI) die chirurgische Genauigkeit erheblich verbessern und zu besseren postoperativen Ergebnissen führen können. Vorteile der Methode sind die präzise Korrektur auf Grad und Millimeter sowie eine verkürzte Operationszeit. Allerdings weisen die Autoren auch auf die zeitaufwendige Planung hin (zwei bis vier Stunden, je nach Komplexität). Zudem wurden die Kosten und der erhöhte Bedarf an Zugängen als potenzielle Nachteile genannt. Die Verwendung von 3D-gedruckten Sägelehren und Schablonen vereinfacht den operativen Ablauf, indem sie eine exakte Vorgabe der Schnittebenen ermöglicht.

Insgesamt bieten 3D-gedruckte, patientenspezifische Implantate einen signifikanten Mehrwert für die moderne Orthopädie und Unfallchirurgie. Ihre zukünftige breite Anwendung hängt jedoch von der Überwindung finanzieller und technischer Herausforderungen ab. Mit fortlaufender Forschung und technologischer Weiterentwicklung könnten diese Implantate kostengünstiger und leichter zugänglich werden, was die klinischen Ergebnissen verbessern und Patientenzufriedenheit steigern würde.

## Resümee

Die Zukunft des 3D-Drucks in der Medizin stellt eine vielversprechende Weiterentwicklung in der präoperativen Planung und der Herstellung von patientenspezifischen Implantaten dar. Der präzise Einsatz von 3D-gedruckten Modellen und individuell angepassten chirurgischen Schablonen bietet nachweislich Vorteile bei komplexen Operationen, insbesondere bei Korrekturosteotomien. Studien verdeutlichen, dass diese Technologie die Genauigkeit chirurgischer Eingriffe erhöht, die Passgenauigkeit verbessert und das Risiko postoperativer Komplikationen wie Instabilität und Fehlstellungen verringert. Herkömmliche Implantate bieten oft nur generische Passformen, während 3D-gedruckte, patientenspezifische Lösungen die exakte Anpassung an die individuelle anatomische Situation ermöglichen. Dies führt zu einer besseren Funktionalität und langfristigen Stabilität.

Ein weiteres spannendes Feld ist die aufkommende Technologie des 4D-Drucks. Gedruckte Implantate erhalten die Fähigkeit, ihre Form oder Funktion als Reaktion auf äußere Reize zu verändern [21, 22]. Diese Strategie könnte eine neue Dimension in der Patientenversorgung eröffnen, da sie die Anpassungsfähigkeit und Funktionalität von Implantaten erweitert. Implantate könnten auf die sich verändernden Bedürfnisse des Körpers reagieren [23]. Die Implementierung solcher dynamischen, reaktionsfähigen Implantate könnte langfristig das Spektrum der Behandlungsmöglichkeiten in der Medizin revolutionieren und personalisierte Therapien auf ein neues Niveau heben.

## Fazit für die Praxis

- Der präzise Einsatz von 3D-gedruckten Modellen und individuell angepassten chirurgischen Schablonen bietet nachweislich Vorteile bei komplexen Operationen, insbesondere bei Korrekturosteotomien.
- Die Genauigkeit chirurgischer Eingriffe wird erhöht, die Passgenauigkeit verbessert und das Risiko postoperativer Komplikationen wie Instabilität und Fehlstellungen verringert.
- Herkömmliche Implantate bieten oft nur generische Passformen, während 3D-gedruckte, patientenspezifische Lösungen die exakte Anpassung an die individuelle anatomische Situation ermöglichen. Dies führt zu einer besseren Funktionalität und langfristigen Stabilität.

## Supplementary Information

Video 1 Korrekturosteotomie am distalen Unterarm
